# Alien Plants Introduced by Different Pathways Differ in Invasion Success: Unintentional Introductions as a Threat to Natural Areas

**DOI:** 10.1371/journal.pone.0024890

**Published:** 2011-09-15

**Authors:** Petr Pyšek, Vojtěch Jarošík, Jan Pergl

**Affiliations:** 1 Department of Invasion Ecology, Institute of Botany, Academy of Sciences of the Czech Republic, Průhonice, Czech Republic; 2 Department of Ecology, Faculty of Science, Charles University, Prague, Czech Republic; 3 Institute of Ecology and Evolution, University of Bern, Bern, Switzerland; Institut Mediterrani d'Estudis Avançats (CSIC/UIB), Spain

## Abstract

**Background:**

Understanding the dimensions of pathways of introduction of alien plants is important for regulating species invasions, but how particular pathways differ in terms of post-invasion success of species they deliver has never been rigorously tested. We asked whether invasion status, distribution and habitat range of 1,007 alien plant species introduced after 1500 A.D. to the Czech Republic differ among four basic pathways of introduction recognized for plants.

**Principal Findings:**

Pathways introducing alien species deliberately as commodities (direct release into the wild; escape from cultivation) result in easier naturalization and invasion than pathways of unintentional introduction (contaminant of a commodity; stowaway arriving without association with it). The proportion of naturalized and invasive species among all introductions delivered by a particular pathway decreases with a decreasing level of direct assistance from humans associated with that pathway, from release and escape to contaminant and stowaway. However, those species that are introduced via unintentional pathways and become invasive are as widely distributed as deliberately introduced species, and those introduced as contaminants invade an even wider range of seminatural habitats.

**Conclusions:**

Pathways associated with deliberate species introductions with commodities and pathways whereby species are unintentionally introduced are contrasting modes of introductions in terms of invasion success. However, various measures of the outcome of the invasion process, in terms of species' invasion success, need to be considered to accurately evaluate the role of and threat imposed by individual pathways. By employing various measures we show that invasions by unintentionally introduced plant species need to be considered by management as seriously as those introduced by horticulture, because they invade a wide range of seminatural habitats, hence representing even a greater threat to natural areas.

## Introduction

The past decade has seen much research focused on practical issues of biological invasions, such as the impact of invasive species [1]–[4], but also on scientific topics generating knowledge that can be used to predict and regulate their introductions [5], [6]. One such topic is research on introduction pathways [7], [8] that are starting to be considered as a powerful instrument of alien species management and biosecurity based on the precautionary principle [9], [10]. Pathways are defined as a suite of processes that result in the introduction of an alien species from one geographical location to another, and vectors include dispersal mechanisms and means of introduction [11]. Good knowledge of both these categories provides options for limiting contamination of vectors (e.g., through control of pest populations in source regions), monitoring pathways for target pests, and generic management measures that may have added benefits beyond the target pest species (e.g., machinery cleaning, contaminant control, hull cleaning and anti-fouling, ballast water exchange). Such interventions have the potential for reducing propagule pressure [12]–[15] and thus the likelihood of establishment and spread. Elucidation of introduction pathways is also crucial for informing various facets of post-incursion management, for example by predicting the genetic diversity of the alien species which has implications for spread and control [16].

Although some early historical sources [e.g.], [ 17,18] provide data-based evidence on the relationship between type of introduction pathway and invasion success [19], the research beyond delivery of a species into a target region, i.e. how species introduced by particular pathways perform in the new regions, has received surprisingly little attention. Insights on whether some pathways are associated with a higher probability of introducing species that will become naturalized or invasive (in the sense of [20]–[22]) in the new region would make it possible to target management measures to those modes of introductions that possess increased invasion risks. Similarly, identifying species traits associated with particular pathways would make it possible to predict threats linked to such pathways, in cases where invasions translate in impact on invaded populations, species and ecosystems [23], [24].

Alien plants are being introduced by a variety of modes, both intentionally and unintentionally [25]–[27] and there is a comprehensive body of information on modes of introduction as well as species traits in national and continental literature and databases [e.g. 25], [26], [28]–[31]. This, together with recently introduced rigorous scheme of pathway classification (8), makes plants a suitable group for detailed analyses of species associated with different pathways.

In this paper we used the alien flora of the Czech Republic, Central Europe, for which comprehensive information exists on various aspects of invasions [28], [32]–[36] to explore (i) how many alien species were introduced by different pathways and whether the pattern of these introductions has changed over time, (ii) whether species introduced by certain pathways are more likely to become invasive, more widespread or occupy a wider range of habitats, and (iii) whether pathways can be related to species traits, i.e. are species with certain traits predisposed to introduction via a particular pathway more so than via other pathways?

## Materials and Methods

### Data analysed

The data set analyzed comprised 1007 neophytes recorded in the flora of the Czech Republic [28]; hybrids were excluded. Each species was classified according to the pathway of introduction into the Czech Republic, using the scheme of Hulme et al. [8], simplified into four categories (not considering two categories: corridors, that are irelevant for plants; and unaided dispersal for which there is not enough data). The following categories were distinguished (note that the sum across pathways exceeds the total number of species analyzed since species introduced by multiple pathways were assigned to each category): (i) introduction by release of species that were directly sown into or planted in the wild; (ii) escape from cultivation; for these two pathways the introduced species itself is the traded commodity; (iii) contaminant refers to species unintentionally introduced with a commodity; (iv) stowaway category includes species unintentionally introduced without association with a commodity. It needs to be noted that in many cases, the category (i) is formally a subgroup of (ii) as species which had been planted in the wild also escape from cultivation, therefore planting in the wild is an important pathway of secondary release following introduction of ornamentals [37], [38]. Categories (i) and (ii) are collectively referred to as deliberate, (iii) and (iv) as unintentional.

Each species was further classified according to its invasion status in the Czech Republic as casual, naturalized but non-invasive, or invasive following Richardson et al. [11], [20], Pyšek et al. [21] and Blackburn et al. [22]; information on the status of species was taken from Pyšek et al. [28]. The distributional characteristics based on the occurrence in the Czech Republic included: (i) Number of grid cells from which the species has been reported, based on the Central-European phytogeographical mapping grid [39] of 10′ ×6′ (longitude × latitude), which at 50° N is 12.0×11.1 km or 133.2 km^2^ (total number of grid cells: 679, available for n = 883 species); (ii) number of habitats in which the species grows (total number of habitat types: 88, n = 276); (data from [40]); and that of (iii) seminatural habitats.

The following traits were assigned to each species (species with multiple trait levels were assigned to each category of a given trait), taking data from the CzechFlor working database held at the Institute of Botany AS CR, based on Pyšek et al. [28]: (i) Taxonomic affiliation (genus, family, order); (ii) Continent of origin, distinguishing species native to the European continent but alien in the Czech Republic = 522, and arrivals from other continents = 667); (iii) Residence time [41], [42], based on the first record of the species outside cultivation in the Czech Republic (available for n = 664 species); (iv) Life form (herb = 753, grass = 119, woody = 135); (v) Life span (annual = 453, perennial = 612); (vi) Life strategy (C, competitive = 541, R, ruderal = 358, S, stress/tolerant  = 139; n = 677 species); (see [43]); (vii) Height (n = 919 species); (viii) Type of reproduction in the Czech Republic (seed only = 282; both seed and vegetative = 225, n = 522 species); (ix) Propagule size (n = 590 species); (x) Dispersal mode (wind = 147, water = 50, animals = 248, self-dispersal = 52, no specialized dispersal = 544; n = 846 species).

### Statistical analysis

#### Frequency of introductions by individual pathways and temporal changes in pathways importance

To test the shape of increase in the cumulative numbers of species introduced by individual pathways, the cumulative numbers were regressed on the years of the species introductions. Before the analyses, the numbers were square-rooted to normalize the data, and the shape of increase first tested by curvilinear polynomial regression, starting with linear regression and adding powers of the years of introduction sequentially until the addition caused nonsignificant reduction in explained variance (e.g. [44]). Because the increases always appeared nonlinear, following Zuur et al. [45: pp. 549–550], they were also tested by generalized additive models. The model best describing the increase was then chosen by comparing the best nonlinear model with the best additive model, using the deletion test. The optimal additive model was assessed by iterations of LOESS smoother (e.g. [46]). The iterations differed in local sensitivity expressed as a span of smoothing between 0–1, where span = 1 corresponds to standard linear regression, with complexity described as equivalent number of parameters (ENP) in curvilinear regression [47]. We started with a small value of span (low smoothing and high proportion of explained variance, [48]) and increased the span slowly to the point at which the LOESS model significantly (P<0.05) differed in ANOVA deletion test from the starting model; a slightly smaller value of span, which did not differ significantly from the starting model, was chosen for final interpretation [49]. Residuals of all models were checked to verify whether they do not show any pattern [50]. Calculations were done in S-PLUS v. 8.1.1 (TIBCO Software).

The differences in rates of introduction of alien species via individual pathways were tested by using inclusion curves, i.e. by plotting the cumulative number of species against years of introduction and comparing the 95% confidence interval (CI) of the times to 50% inclusion [51]–[53]. The rates of introduction among pathways were considered statistically different if the times to 50% inclusion did not overlap in CI. The inclusion curves circumvent the problem of a potential lack of independence between errors in the models, because they represent a type of survival analysis. Calculations were done by employing Fieller's theorem [54], [55: pp. 275–278] in GLIM (version 3.77).

#### Species traits associated with different pathways

To identify whether any species' traits are associated with individual pathways, response variables were scored as yes/no to answer the question of whether or not a species was introduced by a given pathway, and predicted based on the traits. Binary classification trees were used for analyses due to their flexibility and robustness, invariance to monotonic transformations of predictor variables, their ability to use combinations of explanatory variables that are either categorical and/or numeric, facility to deal with nonlinear relationships and high-order interactions, and capacity to treat missing data [56].

The trees were constructed in CART Pro v. 6.0 [57]–[59] by binary recursive partitioning with balanced class weights, assuring that yes/no class for each pathway was treated as equally important for the purpose of classification accuracy. Making a decision when a tree is complete was achieved by growing the tree until it was impossible to grow it further and then examining smaller trees obtained by gradually decreasing the size of the maximal tree [57]. An optimal tree was then determined by testing for misclassification error rates for the largest tree as well as for every smaller tree by ten-fold cross-validation. These cross-validated estimates were then plotted against tree size, and an optimal tree chosen both based on (i) the minimum cost tree rule, which minimizes the cross validated error (the default setting in CART v 6.0; [60], and based on the (ii) one-SE rule, which minimizes cross-validated error within one standard error of the minimum [57]. A series of 50 cross-validations were run [56], and the modal (most likely) optimal tree chosen from six measures of homogeneity of partitioning: Gini, Symmetric Gini, Entropy, Class Probability, Twoing and Ordered Twoing [60], [56]. A modal tree with the chosen best homogeneity measure was then investigated for varying sizes of splitting nodes (2, 5, 10 and 25 cases), and a single optimal tree selected for description. Because classification trees cannot properly handle nested designs such as hierarchical taxonomic levels, to take into account that related taxa can share similar traits (e.g. [61]), the trees were repeatedly constructed including only genera, families and orders [62]. The quality of the chosen tree was evaluated as the overall misclassification rate by comparing the misclassification rate of the optimal tree with misclassification rate of the null model [56], and using cross-validated samples [58] as specificity (i.e. the ability of the model to predict that the pathway is not present when it is not) and sensitivity (the ability of the model to predict that the pathway is present when it is) [63].

The chosen optimal tree, providing an intuitive insight into the kinds of interactions between the predictors, was represented graphically, with the root standing for undivided data at the top, and the terminal nodes, describing the most homogeneous groups of data, at the bottom of the hierarchy. The quality of each split was expressed by its improvement value, corresponding to the overall misclassification rate at the node, with high scores of improvement values corresponding to splits of high quality. In graphical representation, vertical depth of each node was expressed as proportional to its improvement value. Vertical depth of each node thus represented a value similar to explained variance in a linear model. Surrogates of each split, describing splitting rules that closely mimicked the action of the primary split, were assessed and ranked according to their association values, with the highest possible value 1.0 corresponding to the surrogate producing exactly the same split as the primary split. Because categorical explanatory variables with many levels have higher splitting power than continuous variables, to prevent any inherent advantage these variables might have over continuous variables, penalization rules for high category variables [58: p. 88] were applied. Similarly, explanatory variables with missing values have an advantage as splitters. Consequently, these variables were first penalized in proportion to the degree to which their values were missing, and then treated by back-up rules using surrogates that closely mimicked the action of the missing primary splitters [58].

#### Invasion status of species introduced by particular pathways

To test whether there are differences among invasion status of species introduced by different pathways, the total species counts were analyzed by a row × column (i.e., pathway × status) G-test contingency table using generalized linear models with log-link function and Poisson distribution of errors (e.g. [64: pp. 548–550]). To ascertain for which pathways the counts are lower or higher than can be expected by chance, adjusted standardized residuals of the G-test were compared with critical values of normal distribution following Řehák & Řeháková [65]. To assess whether these counts were affected by species' residence times, data on species with known residence times were divided into two subsets, those introduced before (n = 314) and after (n = 350) 1900. The effect of residence time was then tested by the deletion test of two-way interaction of pathway × status × residence time on complex contingency table following Crawley [55: pp. 231–237].

#### Differences in distribution and habitat range among species introduced by particular pathways

To assess whether species introduced by different pathways differ in the number of grid cells and that of habitats occupied we used these two distribution characteristics as the response variables, invasion status (casual, naturalized, invasive) and pathway (release, escape, contaminant, stowaway) as categorical factors, and residence time as a continuous covariate. The total number of habitats and that of seminatural were added as two levels of categorical factors in analyses of habitat range. All these factors and the covariate were considered as fixed explanatory variables. To take into account species relatedness, levels of taxonomic affiliation (genus, family, order) of each species were treated as random explanatory variables. The treatment of taxonomical hierarchy as a random effect means that the inference on taxonomy can be applied to a wider population from which the species are derived, i.e. to any species belonging to that genus, family and order (e.g. [66]).

To reveal optimal models having both fixed and random effects, top-down strategy on linear mixed effect models [67] was applied, following Zuur et al. [45] : pp. 120–129. The modeling started with a beyond optimal model, containing all explanatory variables and their interactions. Using this beyond optimal model, as a first step, the optimal structure of the random component was found. This was done by likelihood ratio (LR) tests on nested models, which were evaluated by restricted maximum likelihood method (REML), obtaining the correct probability values following Verbeke & Molenberghs [68] by testing on the boundary. The nested models included (i) a model without taxonomy (i.e., with no random effect), (ii) with only orders included, (iii) with families within orders, and (iv) with genera within families and orders. If the optimal random component of the model included only genera nested within families and orders, this nested model was further compared with a model including only genera (i.e., without nesting the genera in the taxonomic hierarchy). The results of LR tests on the nested models were confirmed by model selection based on Akaike Information Criterion (AIC).

Once the optimal random structure has been found, as a second step the optimal fixed structure was examined by deletion tests. It was done by maximum likelihood method (ML), keeping the same optimal random structure in all the examined models. Non-significant fixed effects were removed in a step-wise fashion. At each step, the least significant term was removed by LR test from the model, starting with nonsignificant interaction terms. To prevent biases to the model structures caused by correlation between the explanatory variables, all interactions of the same complexity were kept in the model during the stepwise process. In case of significant interactions between factors, the modeling started from the beginning, i.e. from the beyond optimal model, separately for each factor from the significant interaction. These procedures finally yielded minimum adequate models containing only significant factors (i.e., significantly different from zero and from one another) and, at the same time, minimizing AIC (e.g. [64]). As a third step, the final models with the optimal random and fixed structure were presented, using REML.

To normalize data, the numbers of grid cells and habitat types were log- and residence time square-root-transformed before the analyses (e.g. [44]). Fitted models were checked by plotting standardized residuals against fitted values, and by normal probability plots (e.g. [64]). Calculations were done in S-PLUS v. 8.1.1 (TIBCO Software), using the functions *glm* and *gls*. The latter function was applied on models with no random effect, to enable including the models with no random effect in nested LR tests of random components following Zuur et al. [45: p. 122].

### Ethics statement

This paper does not involve field studies – it is analysis of data taken from databases.

## Results

### Frequency of introductions by individual pathways and temporal changes in pathways importance

Among the 1007 neophyte species with available information on the pathway of introduction, 93 (6.7%) were released, 599 (43.1%) are escapes from cultivation, and 443 (31.9%) and 254 (18.4%) were introduced unintentionally as contaminants and stowaways, respectively. The numbers reflect the fact that some species were introduced by multiple pathways.

**Figure 1:**
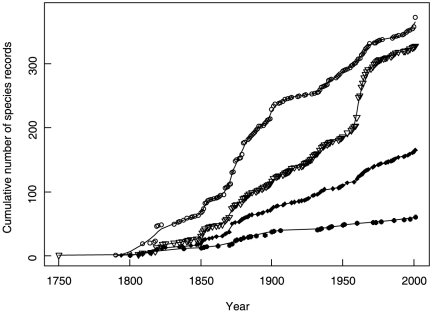
Temporal patterns of introduction of alien neophytes into the Czech Republic with particular pathways. Fitted curves are LOESS best regression models on square root numbers of cumulative records (back-transformed for visualization) with no patterns of residuals, chosen by deletion test against starting models with small spans (<0.1). Release (black circles): span of smoothing = 0.4; equivalent number of parameters in curvilinear regression ENP = 8.2; explained variance r^2^ = 0.99; deletion test against the best polynomial model F = 7.75; df = 1.91; P<0.01. Escape (open circles): span = 0.11; ENP = 27.8; r^2^>0.99; deletion test F = 71.34; df = 1.65; P<0.001. Contaminant (open triangles): span = 0.10; ENP = 31.8; r^2^>0.99; deletion test F = 36.36; df = 5.10; P<0.001. Stowaway (black diamonds): span = 0.10; ENP = 34.0; r^2^>0.99; deletion test F = 45.50; df = 1.57; P<0.001.

There was a steady increase in the cumulative number of species being introduced by individual pathways (Fig. 1) but that of released species decelerated at the beginning of the 20th century. Stowaway species accelerated since 1850, while the pattern for escapes from cultivation indicates increased introduction rates between 1860s and 1900s and that for contaminant between 1960s and 1970s.

Using the mean time to 50% inclusion with 95% confidence interval (CI), the rates of introduction for deliberately introduced species, i.e. released and escaped, were 144.9 (CI = 141.2–148.5) and 146.8 (CI = 146.0–147.6) years, respectively, i.e. shorter than those for unintentionally introduced species, i.e. contaminants (172.9 years, CI = 172.1–173.6) and stowaways (163.8 years, CI = 162.5–165.2). The rates of deliberate introductions did not differ significantly, while among unintentional arrivals stowaway species were being introduced at a faster rate than contaminants.

### Traits of species associated with different pathways

**Figure 2:**
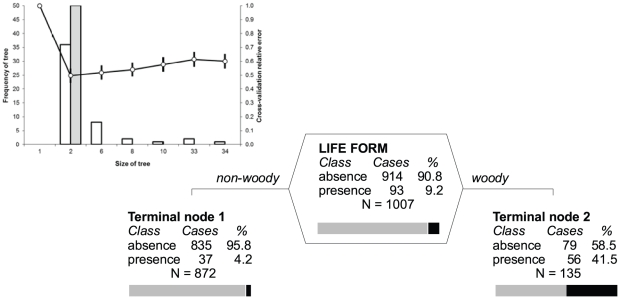
Classification tree analysis of the probability that a species will (presence: black part of the bar) or will not be (absence: grey) introduced by the release pathway. Each node (polygonal table with splitting variable name) and terminal node (with node number) shows a table for presence and absence class, describing the number and percentage of cases in each class. Below the table is the total number of cases (N) and graphical representation of the percentage of presence and absence cases (horizontal bar). For each node, there is a split criterion on its left and right side. Vertical depth of each node is proportional to its improvement value that corresponds to explained variance at the node. Overall misclassification rate of the optimal tree is 11.5%, compared to 50% for the null model; specificity (ability to predict that the pathway is not present when it is not)  = 0.91; sensitivity (ability to predict that the pathway is present when it is)  = 0.59. Inset: Cross-validation processes for the selection of the optimal tree. The line shows a single representative 10-fold cross-validation of the most frequent (modal) optimal tree with standard error (SE) estimate of each tree size. Bar charts are the numbers of the optimal trees of each size (Frequency of tree) selected from a series of 50 cross-validations based on the minimum cost tree rule, which minimizes the cross validated error (white) and one-SE rule which minimizes the cross-validated error within one standard error of the minimum (grey). The most frequent (modal) tree based on both rules has five terminal nodes.

Species released into the wild were more likely trees and shrubs than other life forms; 41.5% of the total number of woody species in the data set were released (Fig. 2, Terminal node 2) compared to only 4.2% among non-woody species (Fig. 2, Terminal node 1).

**Figure 3:**
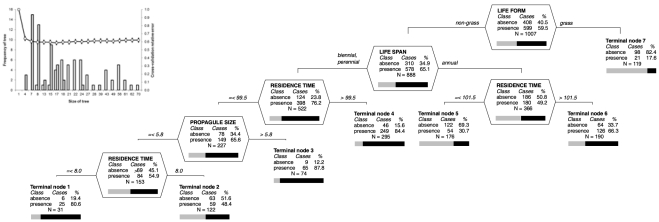
Classification tree analysis of the probability that a species will (presence: black part of the bar) or will not be (absence: grey) introduced by the escape pathway. Overall misclassification rate of the optimal tree is 25.7%, compared to 50% for the null model; specificity (ability to predict that the pathway is not present when it is not)  = 0.63; sensitivity (ability to predict that the pathway is present when it is)  = 0.77. Otherwise as in Fig. 2.

The probability of a species being introduced by the escape pathway was determined by interaction of life form, life span, propagule size and residence time (Fig. 3). It was generally lower for grasses, with only 21.7% of species introduced by this pathway (Fig. 3, Terminal node 7). Among non-grasses, escape introductions were more likely to be perennial (75.2%) than annual (49.2%) but in both life forms the probability increased with longer residence time. Perennials with residence time shorter than ca 100 years were more likely introduced as escapes if they had larger seed (Fig. 3, Terminal node 3).

**Figure 4:**
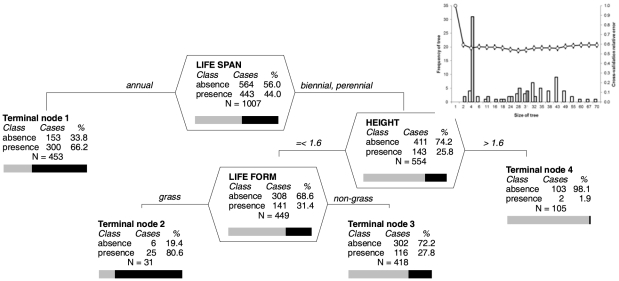
Classification tree analysis of the probability that a species will (presence: black part of the bar) or will not be (absence: grey) introduced by the contaminant pathway. Overall misclassification rate of the optimal tree is 27.5%, compared to 50% for the null model; specificity (ability to predict that the pathway is not present when it is not)  = 0.70; sensitivity (ability to predict that the pathway is present when it is)  = 0.74. Otherwise as in Fig. 2.

The probability that a species will be introduced as a contaminant was high for annuals, 66.2% of which were introduced by this pathway (Fig. 4, Terminal node 1), and among other life forms for grasses shorter than 1.6 m, with 80.6% of such species introduced as contaminants (Fig. 4, Terminal node 2).

Whether a species will be introduced as a stowaway or not is determined by a complex interplay of various traits, including life form (likely for grasses but highly unlikely for woody species) and type of reproduction (more likely for species reproducing only by seed) as major splitters, and fine-tuned by Grime's life strategy, life span, residence time and region of origin at lower splitting levels (Fig. S1).

### Invasion status of species introduced by particular pathways

**Figure 5:**
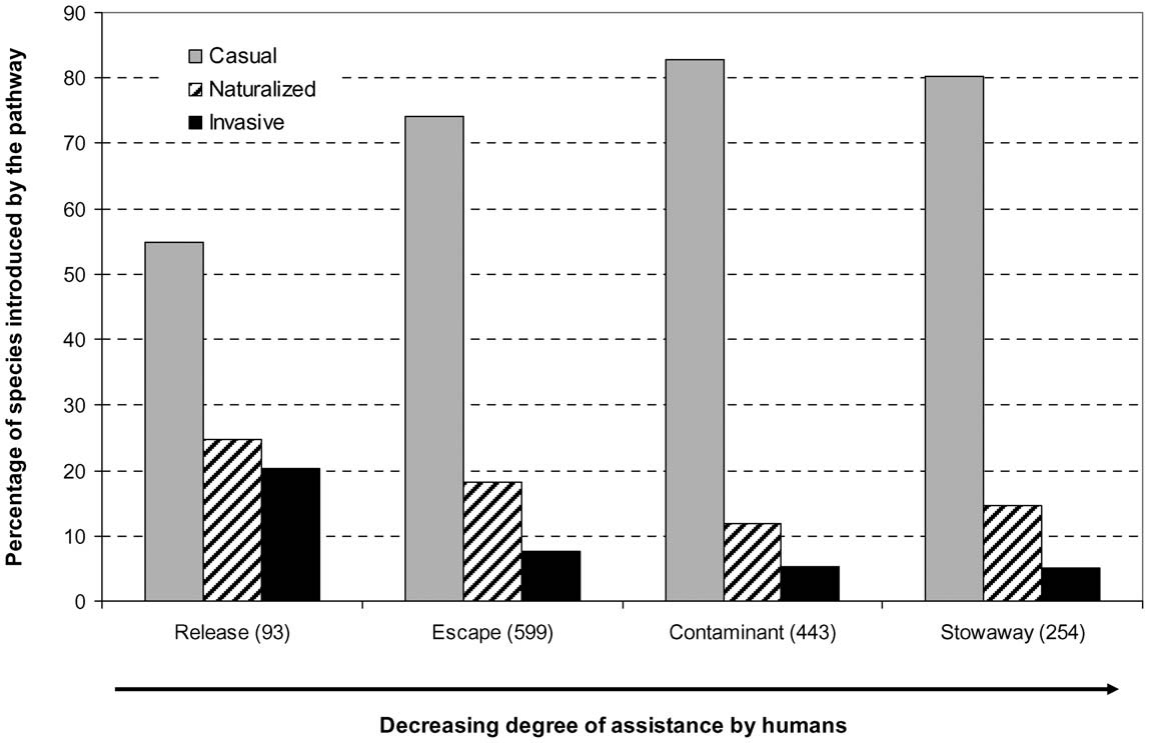
Efficiency of the pathways in terms of species success along the naturalization-invasion continuum (sensu [**13**]). Proportions of casual, naturalized (but not invasive) and invasive species among the total number of plant species introduced via each pathway are shown. Total species numbers are given in parentheses. See Table 1 for statistical analysis.

The four pathways differ strikingly in the number of species introduced that became casual, introduced or invasive (Fig. 5). Deliberate releases resulted in 45.1% of naturalized species among all introduced via this pathway; of those, 24.7% are naturalized but not invasive, and 20.4% invasive. Of the total number of escapes from cultivation there are 25.9% naturalized (18.2% naturalized and 7.7% invasive). The two categories of unintentional pathways, contaminants and stowaway, exhibit values similar to each other, 17.2 and 19.7% of naturalized species, among which there are only 5.2 and 5.1%, respectively, species that became invasive. Deliberate releases thus yielded markedly more invasive species than expected by chance, marginally more than expected of naturalized species and less of casuals. In contrast, species introduced as contaminants were less frequently observed as naturalized than expected, and marginally less than expected as invasive (Table 1). These values were not significantly affected by the species residence time (deletion test of two-way interaction pathway × status × residence time: χ^2^ = 4.571; df = 6, NS).

**Table 1:** Observed and expected counts for invasion status of species introduced into Czech Republic via different pathways.

Pathway of introduction	Invasion status					
	Casual		Naturalized		Invasive	
	Observed	Expected	Observed	Expected	Observed	Expected
Release	51*	71.4	23(*)	14.9	19***	6.8
Escape	444	459.7	109	95.7	46	43.6
Contaminant	367	340.0	53*	70.8	23(*)	32.2
Stowaway	204	194.9	37	40.6	13	18.5

Higher and lower values than expected by chance are marked by asterisks.

(*) <0.1;

* <0.05;

*** <0.001.

### Differences in distribution and habitats among species introduced by particular pathways

Species distribution expressed as the number of occupied grid cells depended, in a different way, on pathways of introduction and invasion status (one-way interaction among pathways × invasion status: LR = 13.01; P<0.05). When casual, naturalized and invasive species were analyzed separately (Table S1), the number of grid cells occupied by casual species resulting from escape increased significantly faster with residence time (regression slope of grid cells on residence time = 0.14) than was the case for other pathways (slope = 0.11; deletion test on a common slope for all pathways: LR = 16.32; P<0.001). Naturalized and invasive species introduced by individual pathways did not differ significantly in the average number of occupied grid cells that increased for all pathways with residence time at the same rate (common regression slope ± standard deviation on residence time: naturalized species = 0.23 ± 0.05; invasive species = 0.20 ± 0.05).

The number of habitats occupied by a species depended on pathway, invasion status and residence time (two-way interaction among pathway × invasion status × residence time: LR = 15.91; P<0.05). For each invasion status evaluated separately, the number of habitats occupied by casual and naturalized species depended on pathways and habitat type considered (one-way interaction pathway × habitat: casuals LR = 86.25; P<0.001; naturalized LR = 45.87; P<0.001), and for invasive species also on residence time (two-way interaction pathway × habitat × residence time: LR = 9.54; P<0.05). For each invasion status and habitat type evaluated separately, the total number of habitats occupied did not differ among pathways and was independent of residence time (Table S2).

For seminatural habitats (Table 2), their number occupied by casual species was independent of residence time, but those casuals that were introduced as released or contaminant were significantly more distributed (on average in 4.0 habitats: the exponential value for the average 1.396 in Table 2) than those as escaped and stowaway (on average in 1.6 habitats; deletion test on the same habitat range for all pathways: LR = 65.11; P<0.001). The number of seminatural habitats occupied by naturalized species was also independent of residence time, but the naturalized species introduced as contaminants were recorded in significantly more seminatural habitats (on average in 8.6) than those introduced by other pathways (on average in 3.9; deletion test on the same habitat range for all pathways: LR = 38.43; P<0.001). The same was true for invasive species; those introduced as contaminants occurred in significantly more seminatural habitats (for zero residence time, i.e. the intercept in Table 2, on average in 4.4 seminatural habitats) than invasive species introduced by other pathways (for zero residence time, on average in 2.9 seminatural habitats; deletion test on the same intercept in all pathways: LR = 26.27; P<0.001). However, unlike for naturalized species, the number of seminatural habitats occupied by invasive species significantly increased with residence time (slope ± SE = 0.07 ± 0.02) and this increase was the same for all pathways (deletion test on different slopes: LR = 2.67; P = 0.44).

**Table 2:** Linear mixed effect minimal adequate models of habitat range.

Source of variation	Invasive status								
	Casual			Naturalized			Invasive		
Random effects	Variance	LR	P	Variance	LR	P	Variance	LR	P
Orders	0.110	12.665	<0.001	0.116	9.808	<0.001	-	-	-
Genera in Orders	-	-	-	0.362	28.908	<0.0001	-	-	-
Genera	-	-	-	-	-	-	0.567	52.929	<0.0001

Fixed effects	Value	Std. Error	df	t-value	P	Value	Std. Error	df	t-value	P	Value	Std. Error	df	t-value	P
Average for contaminant and deliberate	1.396	0.108	85	12.90283	<0.0001	-	-	-	-	-	-	-	-	-	-
Average for escape and stowaway	0.457	0.093^*^	85	-10.104^*^	<0.0001	-	-	-	-	-	-	-	-	-	-
Average for contaminant	-	-	-	-	-	2.150	0.140	40	15.378	<0.0001	-	-	-	-	-
Average for deliberate, escape and stowaway	-	-	-	-	-	1.364	0.091^*^	40	-8.610^*^	<0.0001	-	-	-	-	-
Intercept for contaminant	-	-	-	-	-	-	-	-	-	-	1.480	0.321	32	4.604	<0.001
Intercept for deliberate, escape and stowaway	-	-	-	-	-	-	-	-	-	-	1.055	0.072^*^	32	-5.938^*^	<0.001
Common slope on residence time	-	-	-	-	-	-	-	-	-	-	0.068	0.024	32	2.772	<0.01

Habitat range is expressed as the number of occupied seminatural habitats for casual, naturalized and invasive species in dependence on species taxonomic affiliation (genus, family, order) as random effects, and pathway of introduction (release, escape, contaminant, stowaway) and residence time (years since introduction) as fixed effects. Number of seminatural habitats is log, and residence time square root transformed. Likelihood Ratio (LR) nested models for orders and genera compare nested model with no random effect with model with only orders or genera included. Standard errors and t-values marked by asterisk are values testing difference between the number of occupied seminatural habitats for plants introduced by contaminant and deliberate pathway vs. escape and stowaway pathway (casual species) and introduced as contaminant vs. deliberate, escape and stowaway pathway (naturalized and invasive species).

Except for the significant effect of plant orders for casual species in seminatural habitats (Table 2), the values of variance and likelihood ratio, testing the patterns in species relatedness, appeared always highest for genera (Table 2, Table S1, S2).

## Discussion

### Frequency of introduction by pathways changes over time

Escapes from cultivation were the most important pathway of delivering alien neophyte species to the Czech Republic, accounting for over 40% of all species introduced, followed by accidental arrivals as contaminants that delivered about 30% of species; the contribution of the other two pathways is, in terms of species numbers, less important. This accords with the repeatedly highlighted role of horticulture and the ornamental plant trade [30], [69]–[73].

However, the pattern over time differed among pathways which accords with the fact that the intensity of plant introductions by various pathways reflects dynamics of historical, social and economic events [27], [74] and especially for plants introduced as contaminants, specific singular events such as, e.g., launching a factory processing exotic goods may translate into substantial enrichments of regional floras [75], [76]. The purpose of introduction has also been shown to co-determine the time of arrival of alien species to the Czech Republic on a time scale of centuries, alongside with traits such as species' life strategy and region of origin [32]. Our results illustrate that the two pathways of deliberate introduction that were most likely to deliver invasive species did so at a faster rate than pathways of unintentional introduction. There were, however, some fluctuations in intensity in more or less distant past. Release introductions seem to have levelled off in the first decades of the 20th century, which could reflect the depletion of the pool of species suitable for release into the wild under the local conditions. The course of escape introductions probably reflects the waves of interest in horticulture, with increase at the end of the 19th century, but a marked decline in interest later on which lasted until after the first World War. The dynamics of contaminant introductions rose sharply in the 1960s and 1970s, most likely because of increased research interest in alien plants in the newly founded specialized department of the Institute of Botany AS CR that focused on plants of human-made habitats [77]. However, while individual pathways exhibit specific dynamics, these potential biases do not affect our analysis, which was based on cumulative outcomes of long-term introduction pathways.

### Diversity of introduction pathways contributes to functional diversity of alien floras

Our analysis suggests that individual pathways are associated with specific traits of the species they deliver. Shrubs and trees prevail among released species, reflecting the main purpose of such introductions for landscaping. Perennials owe their association with the escape pathway to their popularity among gardeners [69], [78]. In the latter group of species, those present for longer time were more likely to be recorded as escapes from cultivation, although here the evidence for the role of the amount of time the species was kept in cultivation is only indirect, since the minimum residence time was inferred from the first record in the wild. This interpretation is reasonably safe because it has been previously shown that the length of period in cultivation significantly affects the probability of a species' escape [79]–[81]. Annual life span and grass life form are traits favouring introduction with a commodity, and finally, the introduction as a stowaway is the most opportunistic of all pathways, reflecting the variety of vectors and means of introduction associated with this pathway. This pattern suggests that the complex trait structure of alien floras is a result of multiple introduction processes each contributing not only to species, but also functional diversity of those floras.

### Human assistance translates into invasion success…

Pathways of introductions of alien plants differ strikingly in the magnitude of support provided by humans to species that are translocated to new regions and this fact has consequences in terms of how successful these species will be in overcoming barriers and reaching more advanced stages of the invasion process [20], [22]. Our results show that deliberate pathways in which the species itself is a traded commodity (release, escape) and unintentional pathways (contaminant, stowaway) are two contrasting modes of plant species introductions in terms of invasion success of species delivered. As concerns the escape category, it has been shown that cultivation fosters the success of alien plants, chances of even a small introduced population dramatically increase if it profits from human labour invested [78]. While plants introduced by the release pathway, that are sown or planted into the wild for e.g. landscaping, forestry, bee-keeping etc. [28], [30] may not benefit from creating ideal conditions, sheltering from climatic extremes or assuring reproduction, they are provided with the advantage of direct release into suitable areas, hence a high propagule pressure. This pattern is globally valid; data for Australia show that over 70% of species that become naturalized in 1971–1995 were introduced intentionally [82], the corresponding figure for Florida being 65% [78]. In Germany 50% of the alien flora consists of deliberately introduced species, and more than half of these arrived as ornamentals [83].

Species introduced as contaminants also benefit indirectly from human action as is the case with, e.g. admixture of weed seed to commercially traded seed that can reach enormous densities and benefit from synchronous phenological development with the crops [78]. Therefore, each pathways is associated with some level of human help to species it introduces and our results show that the likelihood that those species will reach more advanced stages of the invasion process decreases with diminishing degree of human direct assistance. The efficiency of the pathway, in terms of delivering species that become naturalized or even invasive, decreases as the assistance of humans becomes less direct, from an alien plant being deliberately released, to it being a commodity, to the assistance reduced to the transport of another commodity, to stowaway not linked to any deliberate movement of commodities (Fig. 1).

### … but there are different measures of invasiveness

However, the supportive role of deliberate pathways ceases to have an effect if other measures of invasion success than invasion status, which is defined as the stage reached by a species along the naturalization-invasion continuum [20], are considered. Unintentional introductions result in species occurring in the same number of grid cells (therefore not constrained in terms of regional occupancy) and occupying the same number of habitats as introductions by the direct-assistance pathways.

However, while direct-assistance pathways result in easier naturalization and invasion, species introduced as contaminants occur on average in more types of seminatural habitats, and this holds true regardless of the stage of invasion these species reach. This suggests that for dispersal and spread in a broad range of seminatural habitats, contaminant species may benefit from the same suite of traits that allowed them to reach the target region without direct human assistance, e.g. namely traits favouring dispersal. Contaminants and stowaways are species that, for introduction, rely on superior dispersal abilities that allow them to become associated with means of human transport from one region to another. That contaminants occupy significantly broader range of seminatural habitats than species introduced as stowaways suggests that being associated with a human-transported commodity represents an additional advantage once the species has been introduced to the target region. This advantage is most likely manifested through systematic spread of a given commodity throughout the country that allows the associated species, regardless of its status, to sample a wider range of habitat types than is the case of species spread more randomly via traffic, human transport or natural features such as water courses. Species introduced as contaminants therefore seem to profit from both aspects of introduction pathways, intentional movement of goods and traits ensuring efficient opportunistic dispersal.

These results also imply that various measures of the outcome of invasion process (in terms of species' invasion success) need to be considered when evaluating the role of and threat imposed by individual pathways; ability to naturalize or invade, and the ability to invade a wide range of habitats and achieve a high regional occupancy tell different stories. This also points to appropriateness of breaking the invasion process down to stages, that, if analyzed separately, provide deeper insights into the nature of invasion process [84]–[86].

### Management implications and research challenges

Understanding pathway efficiency, in terms of the success of species introduced, opens the way to pathway management which is in many instances the best or only way of reducing introductions of new alien species [7], [10]. Only once pathways and vectors of introduction and dissemination are identified, can effective proactive measures be implemented. For instance, the commercial trade in ornamental plants is a major (often the primary) pathway for the introduction and dissemination of invasive alien plants; the most serious plant invaders recruit from garden escapes [30], [69], [87], [88]. Elucidation of the dimensions of this pathway pave the way for a range of interventions, ranging from increasing public awareness of problems, finding alternatives for invasive species [89] and applying biological control, to improving measures of detection and policy enforcement. However, our results imply that while pathways of deliberate introduction indeed result in proportionally more species becoming naturalized or invasive, species arriving via unintentional pathways reach the same distribution, while contaminants colonize an even wider range of seminatural habitats. This implies that serious attention also needs to be paid to pathways where the introduced plant is not a commodity itself. This is further emphasized by the fact that these pathways are associated with high propagule pressure that has been only recently quantified. Lee & Chown [90] have shown that over 1400 seeds of 99 taxa are transported each field season to Antarctica with passenger luggage and cargo, and that 30–50% of these propagules enter the recipient environment. This also points to the allocation of responsibilities for invasions resulting from particular pathways; unlike release and escape where these have been suggested to lay with the applicant and importer, respectively, for invasions resulting from contaminant pathways it is the exporter and for stowaways the carrier who should be held responsible [8]. Another important work related to the quantification of propagule pressure associated with unintentional pathways concerns the role of vehicles as drivers of plant invasions [91]–[92].

Relating traits of introduced species to the probability of them arriving by different pathways opens possibilities for more precise predictions and this knowledge can be incorporated into monitoring and early warning schemes [10]. While the information about pathway efficiency can be used for the pathway management in general, information on the role of species traits associated with pathways could potentially improve screening procedures used for plants and monitoring of potentially dangerous species already in source areas. With respect to predictions, the analysis of phylogenetic relatedness (with the values of variance and likelihood ratio always highest for genera, and genera in most cases represented only by a few species) indicates that generalizations using higher taxonomic levels can be misleading, and assessments of differences in distribution and habitat range among species introduced by particular pathways should be primarily made at a species level, similarly to the assessments of invasion success [36], [85].

Of major importance appears to be research trying to link individual pathways with associated propagule pressure [93]. This seems quite a challenge because usual surrogates such as human population density or economic parameters [13], [72] cannot be used as they are proxies of the total propagule pressure related to a region and are not pathway-related. Some data on propagule pressure associated with individual pathways exist but are mostly related to economic sectors, therefore to direct-assistance pathways, such as ornamental plant trade [69]–[71] or forestry [81]. These studies illustrate the magnitude of propagule pressure associated with escapes, as do scarce data available for release [78], [94]. Much less is known about the amount of propagules introduced by contaminant and stowaway pathways, yet a study addressing this issue resulted in direct management recommendations [95] and our results further indicate that unintentional pathways are associated with the same, or even greater threat as release or escape, because species delivered in this manner are more successful invaders of seminatural habitats.

## Supporting Information

Figure S1 Classification tree analysis of the probability that a species will or will not be introduced by the stowaway pathway.
(DOC)

Table S1 Linear mixed effect minimal adequate models of distribution.
(DOC)

Table S2 Linear mixed effect minimal adequate models of habitat range.
(DOC)
