# Supplementary material for: Alien Plants Introduced by Different Pathways Differ in Invasion Success: Unintentional Introductions as a Threat to Natural Areas
Source: PLoS One. 2011 Sep 15;6(9):e24890. doi: 10.1371/journal.pone.0024890 (PMC3174229; doi:10.1371/journal.pone.0024890)
Supplement: Figure S1 — Classification tree analysis of the probability that a species will or will not be introduced by the stowaway pathway. (DOC) [file pone.0024890.s001.doc]

**Fig. S1. Classification tree analysis of the probability that a species will (presence, ■) or will not be (absence, ■) introduced by the stowaway pathway.** Overall misclassification rate of the optimal tree is 28.7%, compared to 50% for the null model; specificity (ability to predict that the pathway is not present when it is not) = 0.65; sensitivity (ability to predict that the pathway is present when it is) = 0.70. Otherwise as in Fig. 2.
