# Supplementary material for: Alien Plants Introduced by Different Pathways Differ in Invasion Success: Unintentional Introductions as a Threat to Natural Areas
Source: PLoS One. 2011 Sep 15;6(9):e24890. doi: 10.1371/journal.pone.0024890 (PMC3174229; doi:10.1371/journal.pone.0024890)
Supplement: Table S1 — Linear mixed effect minimal adequate models of distribution. (DOC) [file pone.0024890.s002.doc]

**Table S1. Linear mixed effect minimal adequate models of distribution.**

| Source of variation | Invasive status | | | | | | | | | | | | | | | | |
| --- | --- | --- | --- | --- | --- | --- | --- | --- | --- | --- | --- | --- | --- | --- | --- | --- | --- |
|  | Casual | | | | |  | Naturalized | | | | |  | Invasive | | | | |
| Random effects | Variance | | LR | | P |  | Variance | | LR | | P |  | Variance | | LR | | P |
| Orders | 0.005 | | 7.953 | | < 0.01 |  | 0.098 | | 20.004 | | < 0.0001 |  | ̶ | | ̶ | | - |
| Families in orders | ̶ | | ̶ | | - |  | 0.565 | | 8.370 | | < 0.01 |  | ̶ | | ̶ | | - |
| Genera in families in orders | ̶ | | ̶ | | - |  | 2.130 | | 47.896 | | < 0.0001 |  | ̶ | | ̶ | | - |
| Genera in orders | 0.288 | | 35.198 | | < 0.0001 |  | ̶ | | ̶ | | - |  | ̶ | | ̶ | | - |
| Genera | ̶ | | ̶ | | - |  | ̶ | | ̶ | | - |  | 1.432 | | 21.762 | | < 0.0001 |
| Fixed effects | Value | Std. Error | df | t-value | P |  | Value | Std. Error | df | t-value | P |  | Value | Std. Error | df | t-value | P |
| Intercept | -0.189 | 0.118 | 363.000 | -1.599 | 0.1 |  | 0.101 | 0.572 | 70.000 | 0.176 | 0.9 |  | 2.486 | 0.672 | 35.000 | 3.699 | < 0.001 |
| Common slope on residence time | - | - | - | - | - |  | 0.225 | 0.047 | 70.000 | 4.825 | < 0.0001 |  | 0.196 | 0.054 | 35.000 | 3.643 | < 0.001 |
| Slope on residence time for contaminant, deliberate and stowaway | 0.108 | 0.014 | 363.000 | 7.575 | < 0.0001 |  | - | - | - | - | - |  | - | - | - | - | - |
| Slope on residence time for escape | 0.144 | 0.013 | 363.000 | 11.021 | < 0.0001 |  | - | - | - | - | - |  | - | - | - | - | - |

Distribution is expressed as the number of occupied grid cells for casual, naturalized and invasive species in dependence on species taxonomic affiliation (genus, family, order) as random effects, and pathway of introduction (release, escape, contaminant, stowaway) and residence time (years since introduction) as fixed effects. Number of grid cells is log, and residence time square root transformed. Likelihood Ratio (LR) nested models for orders and genera compare nested model with no random effect with model with only orders or genera included.
