# Supplementary material for: Alien Plants Introduced by Different Pathways Differ in Invasion Success: Unintentional Introductions as a Threat to Natural Areas
Source: PLoS One. 2011 Sep 15;6(9):e24890. doi: 10.1371/journal.pone.0024890 (PMC3174229; doi:10.1371/journal.pone.0024890)
Supplement: Table S2 — Linear mixed effect minimal adequate models of habitat range. (DOC) [file pone.0024890.s003.doc]

**Table S2. Linear mixed effect minimal adequate models of habitat range expressed as the number of all occupied habitats.** Otherwise as in Table S1.

| Source of variation | Invasive status | | | | | | | | | | | | | | | | |
| --- | --- | --- | --- | --- | --- | --- | --- | --- | --- | --- | --- | --- | --- | --- | --- | --- | --- |
|  | Casual | | | | |  | Naturalized | | | | |  | Invasive | | | | |
| Random effects | Variance | | LR | | P |  | Variance | | LR | | P |  | Variance | | LR | | P |
| Orders | 0.005 | | 3.822 | | < 0.05 |  | 0.034 | | 5.984 | | < 0.05 |  | ̶ | | ̶ | | - |
| Genera in orders | 0.133 | | 37.768 | | < 0.0001 |  | 0.230 | | 48.561 | | < 0.0001 |  | ̶ | | ̶ | | - |
| Genera | ̶ | | ̶ | | - |  | ̶ | | ̶ | | - |  | 0.406 | | 67.348 | | < 0.0001 |
| Fixed effects | Value | Std. Error | df | t-value | P |  | Value | Std. Error | df | t-value | P |  | Value | Std. Error | df | t-value | P |
| Intercept | 1.294 | 0.049 | 40 | 26.449 | < 0.0001 |  | 1.899 | 0.079 | 41 | 24.141 | < 0.0001 |  | 2.310 | 0.097 | 34.000 | 23.925 | < 0.0001 |
